# Systems Nutrology of Adolescents with Divergence between Measured and Perceived Weight Uncovers a Distinctive Profile Defined by Inverse Relationships of Food Consumption

**DOI:** 10.3390/nu12061670

**Published:** 2020-06-04

**Authors:** Vanessa M. B. Andrade, Mônica L. P. de Santana, Kiyoshi F. Fukutani, Artur T. L. Queiroz, Maria B. Arriaga, Nadjane F. Damascena, Rodrigo C. Menezes, Catarina D. Fernandes, Maria Ester P. Conceição-Machado, Rita de Cássia R. Silva, Bruno B. Andrade

**Affiliations:** 1 Programa de Pós-Graduação em Alimentos, Nutrição e Saúde, Escola de Nutrição, Universidade Federal da Bahia, Salvador 40110-150, Brazil; vanmaggitti@gmail.com (V.M.B.A.); monicalportela@gmail.com (M.L.P.d.S.); naidamascena@hotmail.com (N.F.D.); estercmachado@yahoo.com.br (M.E.P.C.-M.); ritaribeiroufba@gmail.com (R.d.C.R.S.); 2 Multinational Organization Network Sponsoring Translational and Epidemiological Research (MONSTER) Initiative, Salvador 41810-710, Brazil; ferreirafk@gmail.com (K.F.F.); arturlopo@gmail.com (A.T.L.Q.); mbag711@gmail.com (M.B.A.); rodrigocdemenezes@gmail.com (R.C.M.); cati.damasceno@gmail.com (C.D.F.); 3 Instituto Gonçalo Moniz, Fundação Oswaldo Cruz, Salvador 40296-710, Brazil; 4 Curso de Medicina, Faculdade de Tecnologia e Ciências, Salvador 41741-590, Brazil; 5 Faculdade de Medicina, Universidade Federal da Bahia, Salvador 40110-100, Brazil; 6 Curso de Nutrição, União Metropolitana de Educação e Cultura, Salvador 42700-000, Brazil; 7 Universidade Salvador (UNIFACS), Laureate Universities, Salvador 41720-200, Brazil; 8 Escola Bahiana de Medicina e Saúde Pública, Salvador 40290-000, Brazil

**Keywords:** dietary intake, food group, adolescents, dietary patterns, anthropometric status, multidimensional statistical analysis, perception of body weight, divergence between measured and perceived weight

## Abstract

Changes in food consumption, physical inactivity, and other lifestyle habits are potential causes of the obesity epidemic. Paradoxically, the media promotes idealization of a leaner body appearance. Under these circumstances, self-perception of weight by adolescents may be affected. Here, we performed a cross-sectional study, between June and December 2009, to evaluate the interaction between anthropometric status, perceived body weight, and food consumption profiles in 1496 adolescents from public schools in Salvador, Brazil. Data on socio-epidemiological information, anthropometric status, and dietary patterns were analyzed using multidimensional statistical approaches adapted from systems biology. There were dissimilarities between anthropometric status and perception of body weight related to sex. Four dietary patterns were identified based on the food intake profile in the study participants. The distinct dietary patterns were not influenced by divergence between measured and perceived weight. Moreover, network analysis revealed that overestimation of body weight was characterized by a selectivity in ingestion of food groups that resulted in appearance of inverse correlations of consumption. Thus, misperception of body weight is associated with inverse correlations of consumption of certain food groups. These findings may aid individualized nutritional interventions in adolescents who overestimate body weight.

## 1. Introduction

Body image is defined as a subjective view of one’s physical appearance based on self-observation in relation to satisfaction with body size or shape [1]. It is strongly influenced by self-esteem and social acceptance [2,3]. Moreover, during the transitional period of adolescence, there is a strong influence of emotional stress motivated by this same need for acceptance beyond important psychological and physiological changes [4]. Both in adolescence and in adulthood, concern about weight is one of the main causes associated with the negative perception of body image [5]. According to the World Health Organization (WHO), approximately 340 million adolescents are either overweight or obese, making the excess of weight a global pandemic of exponential growth and one of the main comorbidities affecting individuals worldwide [6]. The potential causes are related to physical inactivity, contemporary life habits, and changes in food consumption [7]. During adolescence, there are several determinants of dietary habits, such as meals outside the household, family and social environment, media influence, and other cultural principles [8]. Paradoxically, following the augmented prevalence of obesity, there is mediatic and social pressure for the adoption of a progressively thin and muscular body appearance [9]. In this context, self-perception of weight can be easily affected, potentially leading to psychosocial stress and, consequently, reducing self-esteem while increasing anxiety, depression, and nutritional problems [10]. 

A previous study performed in Brazil demonstrated that up to 38% of the adolescents do not consider their body size normal and more than 15% adopted extreme weight control measures such as use of laxatives, appetite inhibitors, and vomiting inducing drugs [11]. Nonetheless, current dietary habits adopted by adolescents have been characterized as inappropriate, with high total intake of saturated fats, sugars, and salt and low intake of fruits and vegetables [8,12]. These habits tend to perpetuate into adulthood and promote the development of chronic diseases such as hypertension and diabetes [8,12].

A better understanding of the factors that influence dietary habits is important to support measures focused on health improvements in adolescents [13]. We have recently employed a comprehensive analytical method incorporating big data and systems biology tools to evaluate the influence of anthropometric status on the patterns of food consumption [14]. Systems biology uses different information, concepts, and approaches to better understand the whole picture of biological processes and diseases [15]. By incorporating systems biology tools to analyze nutritional data, in what we call ‘systems nutrology’, our previous study reported that overweight and obesity are associated with preferential choices of ingestion of specific food groups [14]. In the present study, we expanded these observations to assess whether the self-perception of the body image influences the pattern of food consumption of the adolescents.

## 2. Materials and Methods

### 2.1. Study Design and Participants

This study is cross-sectional analysis of a databank previously published from our group [14]. The primary study was performed in Salvador, state of Bahia, a large city of Brazil. The subjects were adolescents from state public schools, between June and December 2009. These studies are part of a project that examined psychosocial characteristics that affect health, diet, and cognitive development [10]. The eligibility criteria were: individuals ranging from 11 to 17 years of age, who were attending to school and had parental/guardian approval. Exclusion criteria included diagnosis of eating disorders, psychological conditions such as anxiety or depression, as well as pregnant, nursing, and physical problems that impeded evaluation of anthropometric and/or perception of body weight.

The details on delineation of study sample selection are described elsewhere [14]. Briefly, to define the study sample, we used the simple random sampling technique. The sampling of the study participants was done by conglomerate in two phases: (i) the assortment of the schools, followed by (ii) the selection of the classes. In this phase, the Department of Education of the state of Bahia provided the names and locations of the public schools as well as the list of registered students. These individuals were approached to verify inclusion and exclusion criteria [14], resulting in a total of 1496 students enrolled.

Data on the economic conditions of families were specified by the parents or guardians. To define the economic status for the analyses, we used the criteria provided by the Brazilian Federal Government. Hence, a poor economic status was defined as a monthly average family wage value below $75.00 US dollars whereas a good economic status denoted a wage above $145.00. Standardized and validated surveys [16,17] were carried out to collect data on sex, age, and pubertal development. All the data were recorded using standardized forms—together with information on weight and height, diet assessment, and self-perception of weight—by a panel of dieticians who were trained in interviews and data collection and recording using good clinical practice accredited protocols from the primary research institution leading the study (Escola de Nutrição, Universidade Federal da Bahia, Brazil). 

### 2.2. Assessment of Diet

The food intake was evaluated by means of the semi-quantitative food frequency questionnaire (FFQ), comprising of 97 food elements. The questionnaire used has been designed and validated to infer the reality of the adolescents in Salvador [18]. The FFQ was applied by the same panel of trained dieticians who collected the epidemiological, clinical, and socioeconomic information, during a consultation with each study participant, who described their dietary habits. The frequencies of ingestion of each food element displayed the succeeding choices in response: never/rare; 1–3 times/month; 1 time/week; 2–4 times/week; ≥4 times/week. Furthermore, the consumption of each food and/or preparations was standardized to read units of weight (g) and or volume (mL). This information was next used for computation of the daily ingestion of the food recorded through the FFQ as previously described [14,18]. The results of these calculations were deconvoluted to extrapolate the daily consumption [14,18]. Food elements which were not predicted in the tables were searched via the internet directly on the manufacturer’s website or through recipes. Thus, it was possible to obtain a proxy of the daily total food consumption in grams by calculations based on weekly and monthly consumption. For the statistical analysis of food consumption, the 97 food elements that composed the FFQ were gathered according to similarity in nutritional composition and dietary lifestyles of the population of the Northeast of Brazil, which resulted in 14 food groups that are described in Table S1 and in [14].

### 2.3. Evaluation of Anthropometric Status

Weight was assessed using a digital scale (Master Balanças, Goiania, Brazil), whereas height was measured using portable stadiometer (Seca, Hamburg, Germany). For the analysis, we subtracted the value of the weight of the uniform (100 g). The body mass index (BMI) was assessed following the reference tables from the WHO established in 2007. Percentiles of the BMI values were considered to establish the anthropometric status by sex and age [19]. The different anthropometric statuses were defined as follows: underweight (<3rd percentile), normal weight (≥3rd percentile and <85th percentile), overweight (≥85th percentile and <97th percentile), or obese (≥97th percentile) [14]. 

### 2.4. Self-Perception Assessment and Divergence between Measured and Perceived Weight

Self-perception of anthropometric status was identified by questioning “How do you feel about your weight?” The response choices were ‘thin’, ‘very thin’, ‘normal’, ‘fat’, or ‘very fat’. To perform the statistical statistics, three groups were created: (i) thin/very thin; (ii) normal; (iii) fat/very fat. This information was crossed with the assessment of the anthropometric status categories, to create a variable ‘divergence between measured and perceived weight’ (DMPW) measured perceived weight divergence. DMPW was created by comparing measured and classified BMI according to WHO [19] and self-perceived weight by adolescents. When there was agreement between the measured and perceived anthropometric status, the adolescents were considered to be concordant (“Agreed”) [13,20]. Underestimation was considered when adolescents perceived themselves as eutrophic or underweight but were classified as overweight/obese or eutrophic, respectively [13,20]. Finally, overestimation was considered when adolescents perceived themselves as eutrophic or fat, but were classified as thin, or adolescents perceived as fat and were classified as eutrophic [13,20]. For the purpose of the analyzes, individuals who agreed between their measured and perceived weight were used as reference.

### 2.5. Statistical Analysis

Descriptive statistics were presented to describe the study participants. We tested the continuous variables for Gaussian distribution using the D’Agostino–Pearson test, and no variable was parametric, except for abundance of consumption of the food groups. Consequently, medians and interquartile ranges (IQR) were employed to represent central tendency and dispersion. Distribution and dispersion of the values of abundance of consumption were presented as mean and standard deviation (SD). All comparisons were pre-specified. The Mann–Whitney *U* test was used to compare variables between two groups whereas the Kruskal–Wallis test with Dunn’s multiple comparisons ad hoc test was utilized to compare >2 groups. For comparisons of abundance of food consumption, the one-way ANOVA was used. Categorical variables were displayed as frequency (%) and compared using the Pearson’s chi-square test. 

### 2.6. Systems Nutrology Analysis

To evaluate the overall profile of consumption of the different food groups in the sample, data on total consumption in grams was transformed into abundance of consumption relative to total diet. This method has been commonly used in ecological analysis, in which proportion of each species is calculated to estimate representativeness [21,22]. Herein, this approach led us to define the proportion of consumption of each food or food group in the total ingestion of all the food groups (e.g., the percentage of the total ingestion that is represented by each food group). Next, to define the dietary patterns, an unsupervised hierarchical cluster analysis was performed (Ward’s method). Hence, individuals and abundance of food consumption were both grouped based on similarity. For this analysis, a color map was built with values of abundance of consumption for each food group. The different clusters were defined by overall similarity of food consumption, represented using dendrograms denoting the Euclidean distances [23]. We used bubble plots to illustrate the representativeness of consumption of each food group in each one of the food patterns calculated. Pie charts were used to illustrate frequency of individuals grouped per sex, anthropometric status, and DMPW status in each dietary pattern. 

To test for association between DMPW and the different dietary patterns identified (*n* = 4) in the hierarchical grouping approach, we performed a multinomial multivariate logistic regression analysis, adjusted for sex and anthropometric status [24]. In this approach, the outcome variable was the pattern of food consumption and the main exposure variable was DMPW. In addition, the dietary pattern 2 was used as the baseline for estimation of odds of consumption of the other patterns, given that: (i) there was no clear predominance of any food group in abundance of consumption; and (ii) the average values of abundance of consumption of each food group in the diet were the lowest in general when compared to the other patterns. For these analyzes, a statistical resource was also used to weight the data in relation to the sampling method of the study (by conglomerate). Data were weighted to consider the recruitment delineation and estimate the design effects (Deff). In this approach, Deff values above 3 usually mean that there is a high degree of intra-conglomerate homogeneity and therefore the association tested may have been influenced by the type of sampling. On the other hand, low Deff values indicate that there was little influence of sampling on the association [25].

Correlations between total intake in grams of each food group were evaluated by the Spearman rank test as recently described [14]. We created correlation matrices for each subdivision of study participants. We next performed 100× bootstrap in each matrix [26,27]. This statistical approach was performed estimate in a more realistically manner the distribution of the correlations per study group, while simultaneously addressing the issue of multiple measures and/or comparisons by means of random sampling techniques. This approach is a commonly applied in multidimensional analyses to result in more significant reliability of the findings. In this study, the following criteria was used to include the detected correlations in the network analyses: (i) correlations with *p*-values < 5%; (ii) correlations with Spearman rank (rho [r]) values > ±0.5; and (iii) correlations that persisted with significant *p*-values and rho values in a minimum of 50% of the 100 bootstraps. The network densities were calculated according to the formula: *L*/(*N* × (*N* − 1)/2), with *L* representing the number of significant correlations (criteria defined above) and *N* accounting for the number of nodes (food groups). Of note, the network density denotes the number of significant correlations within the total conceivable number of correlations in each matrix [14]. The network density values were compared between groups using the Kruskal–Wallis test with Dunn’s multiple-comparison ad hoc test. Finally, we performed a node analysis in the networks, which quantified the number of statistically significant correlations identified for each food group, detected in each one of the main subgroups of study participants.

Sample size calculations indicated that 50 individuals per group would result in a study power of 90% and alpha error value of 1%, to detect at least 2-fold-variation in ingestion of at least 1 food group among the different groups by DMPW status. Statistically significant differences were defined by a *p*-value < 5% after adjustment for multiple comparisons using the Holm–Bonferroni’s method. The calculations and figure visualization were performed using the following applications: GraphPad Prism 8.0 (GraphPad Software, La Jolla, CA, USA), JMP 14.0 (SAS, Cary, NC, USA), and R 4.0 (R Foundation, Vienna, Austria). 

### 2.7. Ethics Statement

The study was approved by the Ethics and Research Committee of the Institute of Collective Health of the Federal University of Bahia (protocol no. 002-08CEP/ISC). Written informed consent was obtained from all participants or their legally responsible guardians, and all clinical investigations were conducted according to the principles expressed in the Declaration of Helsinki. 

## 3. Results

### 3.1. Characteristics of Participants

The majority of the study participants was composed by females (57.1%), with median age of 14.3 years (IQR: 13.1–15.5). Moreover, individuals were mostly eutrophic (77.2%), whereas 8.8% were overweight and 5.9% were obese (Table 1). Information on the economic conditions of families was provided by the parents. The study sample included roughly equal distribution in both poor and good economic status, with no statistically significant differences between the subgroups of individuals divided by DMPW status (*p* = 0.1347; Table 1). Assessment of pubertal development revealed a preponderance of the individuals at post-pubertal stage (69.6%), followed by pubertal and pre-pubertal stages (Table 1). When the study participants were stratified according to DMPW status, we observed that most of participants agreed between measured and perceived weight (*n* = 1022, 68.3%), whereas 19.7% (*n* = 294) underestimated and 12% (*n* = 180) overestimated their weight (Table 1). The subgroups of individuals with different DMPW status were similar with regard to age (*p* = 0.4383), socioeconomic status (*p* = 0.1347), BMI values (*p* = 0.7086), and pubertal development (*p* = 0.1828; Table 2). Furthermore, we detected a higher frequency of girls who overestimated and boys who underestimated their anthropometric status (*p* < 0.001, Table 1).

### 3.2. Association between Anthropometric Status and Divergence between Measured and Perceived Weight

**Figure 1:**
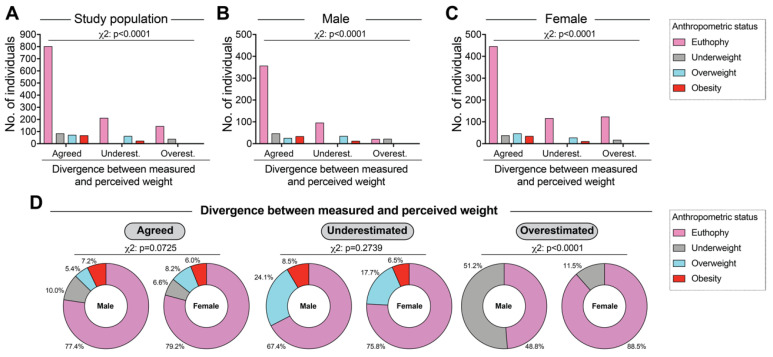
Frequency distribution of divergence between measured and perceived weight, stratified by anthropometric status and sex. (**A**) in all the study participants. (**B**) Male sex. (**C**) Female sex. (**D**) Frequency of different anthropometric status strata in study participants grouped according to divergence between measured and perceived weight. Data were compared using the Pearson’s chi–square test.

Additional analyzes were performed to better describe the frequency of individuals regarding the different categories of DMPW. Thus, it was possible to observe in the total study sample that the majority of individuals were eutrophic regardless of the DMPW status (*p* < 0.0001, Figure 1A). Such predominance of eutrophic individuals was detected in both male and female participants (Figure 1B,C). In addition, it was observed that the frequency of adolescents with obesity was higher in the group of those who agreed with their weight compared with their discordant peers in the total sample (chi square *p* < 0.0001; Figure 1A) and also among male (Figure 1B) and female participants (Figure 1C). Furthermore, we identified that overestimation of the anthropometric status was present only among those with eutrophy or thinness in (Figure 1A–C). We detected no difference in the frequency of the diverse anthropometric strata between male and female participants who agreed in their measured and perceived weight (*p* = 0.0725, Figure 1D, left panel) and also among those who underestimated their weight (*p* = 0.2739, Figure 1D, central panel). However, as shown in Figure 1D (right panel), among the study participants who overestimated their weight, slightly more than half of the boys were underweight (51.2%), whereas the vast majority of the girls were eutrophic (88.5%), highlighting discrepancies related to sex (*p* < 0.0001).

### 3.3. Dietary Patterns 

**Figure 2:**
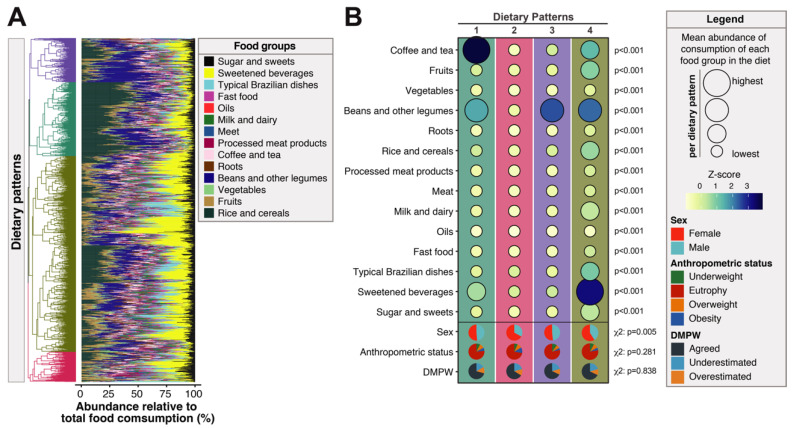
Profiles of consumption of food groups using hierarchical clustering. The abundance of consumption of the indicated food groups in the diet was calculated for each individual as described in Methods. (**A**) Hierarchical cluster analysis (Ward’s unsupervised method), in which the dendrograms represent the Euclidean distance, was used as an approach to identify different dietary patterns. Using this approach, it was possible to identify four major consumption patterns. (**B**) Upper panel shows the average relative abundance consumption of each food group within each dietary pattern identified in the hierarchical cluster analysis. Lower panel shows frequencies individuals in each dietary pattern stratified by sex, anthropometric status and also by divergence between measured and perceived weight (DMPW). The average relative abundance of consumption of each food group was compared between the different dietary patterns using one-way ANOVA. Proportions of sex, anthropometric status, and divergence between measured and perceived weight were compared between the different dietary profiles using the Pearson’s chi-square test. All *p*-values are indicated.

The study participants received a standardized and validated questionnaire to investigate the frequency of food consumption. Primary analyses revealed no statistically significant differences in total consumption of food or food groups in grams between the groups of individuals stratified according to the DMPW (Table 2). To identify dietary patterns, the representativeness of consumption of each food group in the overall diet of each individual was calculated (see Methods for details), represented here as abundance relative to total food consumption (Figure 2A). With these values, a hierarchical cluster analysis was performed to identify groups based on similarity of consumption. This approach revealed four major patterns of food consumption. The distinct dietary patterns were very different in terms of abundance of food group consumption (Table 3). The dietary pattern 4 was the one with the highest values of abundance of consumption of each food or food group whereas the pattern 2 in general exhibited the lowest values of abundance (Table 3). Furthermore, the dietary patterns also differed in terms of each food group was more representative of the total food consumption (Figure 2B). In the dietary pattern 1, ‘coffee and tea’ was the group with the highest abundance of consumption, followed by ‘beans and other legumes’ and ‘sweetened beverages’. Dietary pattern 2 did not have predominance of consumption of any food group. Pattern 3 had sole predominance of ‘beans and other legumes’. Finally, pattern 4 displayed more balanced distribution of abundance of food consumption, with the highest values being detected for ‘sweetened beverages’, followed by ‘beans and other legumes’, ‘coffee and tea’, ‘fruits’, ‘rice and cereals’, and ‘typical Brazilian dishes’, and other groups with lower values (Figure 2B). Additional analysis indicated that female adolescents, eutrophic respondents, and those who agreed between measured and perceived weight were those who consumed more of all food profiles. Among the different dietary profiles, female participants were more frequent in the patterns 2 and 4 (*p* = 0.005, Figure 2B). No preferential dietary pattern was found between individuals presenting with the diverse anthropometric statuses (*p* = 0.281) or stratified according to DMPW (*p* = 0.838) (Figure 2B).

**Figure 3:**
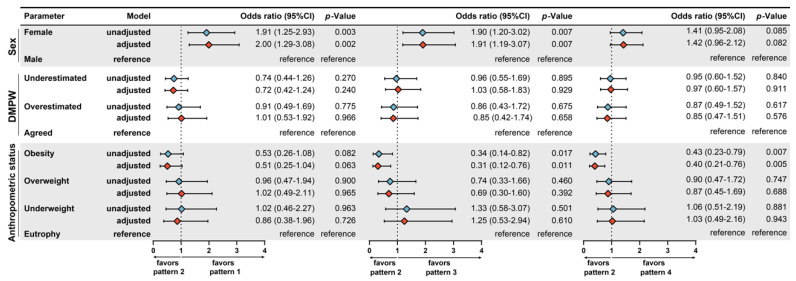
Multinomial logistic regression to test independent associations with the distinct dietary patterns. Adjustment was performed for all variables presented in the figure. The dietary pattern 2 profile was used as reference to test associations between variables and patterns 1, 3, or 4. The statistical significance was estimated through a multinomial logistic regression model. The results were also weighted for study design effect (Deff). The variable dietary pattern displayed low degree of intra-conglomerate heterogeneity (Deff = 1.12). Thus, the study design had low impact on the variance of food pattern values in the study participants. Abbreviations: CI—confidence interval; DMPW—divergence between measured and perceived weight.

Furthermore, we used a multinomial logistic regression model to test independent associations between the DMPW and the distinct dietary patterns. In this approach, the outcome variable was the pattern of food consumption and the main exposure variable was DMPW, with adjustment for sex and anthropometric status. In addition, the dietary pattern 2 was used as the baseline for estimation of odds of consumption of the other patterns (see Methods for details; Table 3 and Figure 2B). The regression analysis demonstrated that DMPW was not associated with any dietary pattern. Conversely, the variables used in the adjustment did show association with dietary patterns: obese individuals were less likely to adhere to dietary patterns 3 and 4, whereas female participants more frequently adhered to the patterns 1 and 3 (Figure 3). 

### 3.4. Network Analysis of Food Consumption

**Figure 4:**
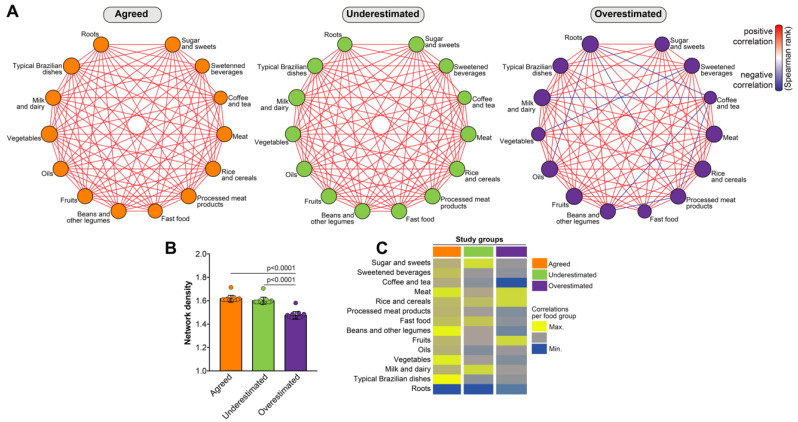
Network analysis of food consumption reveals negative correlations in individuals who overestimated their weight. (**A**) Spearman correlation matrices were designed for each study subgroup. Each matrix was submitted to 100× bootstrap. Correlations with adjusted *p*-values < 0.05, with rho(r) values > ±0.5 and which persisted exhibiting such values significant in at least 50% of the bootstraps were included in this analysis. (**B**) The network density, denoting the number of correlations (see Methods for details), was compared between using the Kruskal–Wallis test with Dunn’s multiple comparisons post-test. (**C**) Node analysis illustrate the number of correlations for each food group in the distinct subcategories of DMPW.

The results described so far have revealed no clear association between DMPW and adherence to a specific dietary pattern. To promote health, it is thought that the ideal diet includes a balanced ingestion of a diverse range of food groups. In this context, we have recently published that network analysis of the correlations between the ingestion of the different food groups could highlight a distinct profile that could hallmark overweight and obese adolescents [14]. This published approach differs from the dietary pattern analyses reported in our results above because it directly examines whether elevated consumption of a given food group is followed by increases or decreases in ingestion of other groups, highlighting dietary predilections of subcategories of adolescents [14]. Here, we used information on total consumption (grams) of each food group to build networks based on correlation matrices and compared the subgroups of individuals grouped according to the DMPW status. We observed that the group of participants who agreed between measured and perceived weight exhibited a large number of correlations (Figure 4A, left panel). Of note, all the statistically significant correlations detected in this study group were positive, inferring that the augmented ingestion of a given food group was associated with increased ingestion of additional groups. A comparable arrangement of correlations was detected in the group of participants who underestimated their weight (Figure 4A, central panel). Strikingly, several negative correlations, meaning inverse trends in consumption of different food groups, were observed in the group of adolescents who overestimated their weight (Figure 4A, right panel). Indeed, consumption of roots, for example, was inversely correlated with consumption of ‘oils’, ‘processed meat products’, and ‘coffee and tea’, whereas intake of ‘vegetables’ was negatively related to that of ‘sweetened beverages’ (Figure 4A, right panel). 

Furthermore, we compared network densities between the groups of individuals stratified according to DMPW [14]. This approach let us to identify the food groups which ingestion was mostly correlated with that of the other dietary groups examined. We found that individuals who overestimated their weight exhibited the lowest network density values (*p* < 0.001, compared to each one of the other groups, Figure 4B). These findings argue that qualitative (whether a correlation is positive or negative) and quantitative (quantity of significant correlations detected) discrepancies are observed in dietary habits of individuals who overestimated their weight. We next performed an analysis to identify the food groups that most participated in the networks by means of having its ingestion correlated with that of other food groups, [14]. This analytical approach revealed a predominance of ‘typical Brazilian dishes’, ‘vegetables’, ‘beans and other legumes’, and ‘meat’ in the group of individuals who agreed between their measured and perceived weight, indicating that the consumption of these food groups was directly proportional to intake of several other food groups (Figure 4C). In the group of adolescents who underestimated their weight, the most relevant food groups in the network were ‘milk and dairy’ and ‘sugar and sweets’ (Figure 4C). Lastly, ‘fruits’, ‘rice and cereals’, and ‘meat’ were the most significant nodes of the networks from those who overestimated their weight. These analyses characterized qualitative and quantitative associations of consumption between distinct food groups, emphasizing disparities linked to DMPW.

## 4. Discussion

During adolescence, several factors influence both the dietary habits and the self-perception of the body image, such as social media, family environment, industrialized meals, and cultural principles [1,9,28,29,30]. It is important to understand the contribution of such factors on the dietary habits, in order to aid establishment of better dietary interventions to promote health in such sensible population. In the study presented here, two collaborative research groups—with distinct expertise, clinical nutrition, and multidimensional statistics—joined efforts to try to deconvolute the dietary profiles of a large number of adolescents from Brazil, which is experiencing a substantial increase in prevalence of overweight. We employed a novel comprehensive analytical approach using big data and systems biology tools to extensively describe the dietary patterns and food consumption profiles of 1,496 adolescents from the public-school system in Northeast Brazil. In addition, we examined the potential association between food consumption profiles, anthropometric status and DMPW. Such an analytical approach is innovative and is part of a new study field we would like to denominate ‘systems nutrology’. Research from such a new field may expand understanding about the nuances of interactions between factors that underly dietary behaviors. 

Our initial analyses revealed that, despite the fact that the majority of the studied adolescents showed agreement between the measured and the perceived weight, the total frequency of DMPW was relatively high (31.4%). It is worth noting that these results are comparable to what has been reported by other studies in South Korea (49.3%) [31], United States (25.5%) [32], Italy (27.6%), and Pakistan (42.4%) [20,33]. In 2007, another investigation carried out in Salvador, Brazil, the same city of the present study, which included children and adolescents enrolled in private schools, identified a DMPW frequency as high as 35.2% [34]. These findings indicate that DMPW is indeed a common observation and its implications should be investigated. After stratifying our study participants according to sex, it was noticed that the global profile of DMPW was different between boys and girls. Our results demonstrated that female adolescents more often overestimated their weight whereas male adolescents showed a higher frequency of underestimation. The frequency of eutrophic adolescents was higher in the group in which there was agreement between measured and perceived weight. Interestingly, among the persons that overestimated their weight, female individuals were more frequently eutrophic, whereas males were more frequently underweight. These results are in line with a North American study of more than 6500 teenagers [35], and another investigation of 1,643 teenagers from Italy and more recently in Thailand [33,36]. Thus, there is strong evidence to support that there are important differences in self-perceived weight between the sexes and that female individuals tend to overestimate their weight. 

The fact that many students who overestimated their body weight were in fact eutrophic or even underweight reinforces the idea that there is social pressure for the individual to fit the patterns for thinness. This observation may be related with the fact that puberty changes physical appearance, weight, and fat distribution, which can promote dissatisfaction with weight, especially among girls [29,37]. Adolescence is a period of constant concern with body image, precisely because of these physiological changes that occur with the body. It is also worth noting that it has been reported that teenagers with adequate weight who perceived themselves to be overweight tend to gain weight when compared to adolescents who do not perceive themselves to be overweight, and among girls this relationship is stronger than in boys [9]. This information is in direct alignment with our results, as described above.

Several studies have shown that adolescents, and especially female, who are concerned about being overweight, regardless of their actual weight, are more likely to adopt extreme practices for weight control and restricted diets, and tend to suffer more from depression and low self-esteem, predisposing them to develop nutritional disorders or even to develop eating disorders, such as anorexia and bulimia nervosa [13,38]. A previous study from North America examined 5018 teenager girls to test associations between weight status, perceived weight, and health-related quality of life (HRQOL) [30]. This study demonstrated that the perception of being overweight was associated with worse physical, emotional, academic, and social HRQOL score values [30]. Therefore, it is important to better understand the pattern of food consumption of persons with DMPW in order to implement a more efficient intervention on their food habits and consequently improving their quality of life. Of note, our study was not designed to investigate nuances between male and female adolescents that may underlie the differences in self-perception of weight and drive DMPW. The evidence provided from previous studies and discussed above argue that female adolescents may have increase susceptibility to develop psychological disorders which in turn favor the occurrence of DMPW and the onset of dietary disorders. Future studies focused on the elucidation of these potential links are warranted to test this hypothesis.

To estimate the dietary patters of our study participants, we employed a method adapted from ecology studies [21,22]. By transforming the total consumption of each food group (annotated in grams) into abundance of consumption in the diet, it was possible to infer the representativeness of ingestion of a given food group relative to total diet. A hierarchical cluster analysis grouped individuals based on their profile of abundance of consumption of the different food groups and identified four major dietary patterns. Such patterns differed from each other in terms of abundance of consumption of each food groups and also by the food groups that were more predominant in the diet. The composition of dietary patterns varies according to the location and cultural and social epidemiological characteristics of the adolescents studied, in addition to being greatly influenced by the analytical methodology employed [39]. Another issue that can influence the standard dietary composition is a very common food preference in this stage of adolescence, marked by low consumption of foods from groups of milk and dairy products, fruits, vegetables, meats, oils and fats, and roots, which may be associated not only with access and availability but also with that preference. Studies related to food consumption among adolescents have shown low consumption of fruits, vegetables, milk and dairy products, and protein sources [12,40]. Our analyses also indicated that female adolescents who were eutrophic and who agreed between measured and perceived weight were those who consumed more of all the dietary patterns. 

Importantly, no preferential dietary pattern, as assessed by total food consumption in grams or by abundance of consumption, was found between individuals being part of the distinct DMPW subgroups. These results were validated by a multinomial logistic regression analysis. Thus, the average consumption of food groups was not influenced by self-perceived weight. Analyses of consumption of food groups in grams, and also by means of abundance/representativeness in total diet, do not provide much detailed information of balance of intake. In this scenario, the correlation analyses performed here could defined peculiarities in the relationships between habits of food ingestion. Such analytical approach has been previously reported in nutrition [41], and we have recently employed network visualization of statistical interactions [14], which have been more frequently reported in transcriptomic [42], and immunologic analyses [26,27]. In the present study, this approach was once again capable of underscoring associations which have not yet been acknowledged by other statistical methods. Hence, the Spearman correlation networks revealed that: (i) several significant correlations in food consumption were detected between the diverse food groups in each subcategory of individuals according to the DMPW, denoting the dietary behavior of each adolescent is a synchronized process that results in concurrent ingestion of a number of other distinct food groups; (ii) among those who agreed between their measure and perceived weight and also in those who underestimated their weight, only positive correlations were detected, demonstrating that increases in ingestion of a given food group is directly followed by augmented consumption of other groups; (iii) adolescents who overestimated their weight exhibit a number of negative associations among the consumption of food groups, determining that there is preferential intake of specific food groups compared to others. It should be highlighted that, in our previous study of this population [14], we reported that negative correlations in ingestion of food groups were observed in individuals with overweight and obesity, whereas only positive correlations were found in eutrophic and underweight adolescents. We concluded that weight gain is accompanied by preferential choice of ingestion of specific food groups; a phenomenon that was not observed in eutrophic individuals. Therefore, dietary food selection has potential to result in a nutritional imbalance that leads to weight gain. Together with the results presented here, we hypothesize that an impression of excessive weight, even in eutrophic individuals, is sufficient to trigger a behavior associated with the selective choice of food consumption similar to what is observed in individuals with overweight and or obesity. Reinforcing this idea, a recent study which examined weight status and weight misperception among 10,708 Chinese children and adolescents has suggested that weight misperception may be an independent risk factor of unhealthy lifestyles and that this could accelerate gain of weight [43]. Altogether, these observations reinforce the need for additional studies to test whether specific patterns of food consumption can interfere on the change of abnormal eating habits. The present study brings an innovative approach to visualize consumption profiles and its association with self-perceived weight in teenagers, being able to potentially direct a nutritional intervention focused on normalizing such relationships of consumption of food groups.

The present study has some limitations. We employed hierarchical cluster analysis to define the dietary patterns, as such an approach has the advantage of delivering a sharp depiction of precisely what groups of individuals are consuming. In this analysis, since each individual is allocated to a single cluster, and such cluster is characterized in terms of abundance of consumption of each food group, then it makes easier to define nutritional deviations and help implementing dietary interventions. A limitation of this analysis is the low power to perceive associations with clinical outcomes in situations where the study has a small sample size. Nevertheless, our study power and sample size calculations demonstrated that this is not the case of the study presented here. Furthermore, the FFQ is not a tool with 100% reliability because it is dependent on memory or capacity to report. In addition, FFQ does not record consumption of dietary groups not listed in the questionnaire, leading to potential sub-notification of food ingestion. Finally, the different subgroups of individuals with distinct DMPW statuses could influence the capacity to find statistically significant correlations. For this reason, we used correlation matrices submitted to 100× bootstrapping, which performs re-sampling of subgroups and compensate for discrepancies in sample size for the network analysis, as described in Methods. Notwithstanding, the results described in the present study are strong since they are a consequence of a mixture of detailed clinical and nutritional investigation of a large number of adolescents, as well as use of powerful statistical analyzes that, when combined, delineate the specifics of the dietary consumption of the adolescents related to the DMPW status.

## 5. Conclusions

Our findings reveal that overestimation of weight in adolescents is related to preferential choices of consumption of certain food groups, which results in the presence of inverse correlations visualized in Spearman networks. Such behavior is nonexistent in those who agreed between measured and perceived weight, and also in adolescents who underestimated their weight. These results might help nutritional interventions focused on the adolescents with DMPW, consequently helping to improve their quality of life and mental health and preventing them from developing nutritional disorders. 

## Figures and Tables

**Table 1:** Characteristics of the study participants.

Characteristic	Total *n* (%)	Agreed *n* (%)	Underestimated *n* (%)	Overestimated *n* (%)	*p*-Value
N	1496	1022	294	180	
Age-years median (IQR)	14.3 (13.2–15.5)	14.3 (13.2–15.3)	14.5 (13.2–15.6)	14.3 (13.4–15.7)	0.4383
Sex					<0.001
Female	854 (57.1)	562 (55)	153 (52)	139 (77.2)	
Male	642 (42.9)	460 (45)	141 (48)	41 (22.8)	
Socioeconomic status *					0.1347
Good economic condition	727 (48.6)	506 (49.5)	129 (43.9)	92 (51.1)	
Poor economic condition	721 (48.2)	479 (46.9)	158 (53.7)	84 (46.7)	
BMI-Kg/m^2^ median (IQR)	18.9 (17.2–21.0)	19.0 (17.2–20.8)	18.4 (17.0–21.6)	19.6 (17.1–21.3)	0.7086
Pubertal development *					0.1828
Pre-pubertal	126 (8.4)	87 (8.5)	31 (10.5)	8 (4.4)	
Pubertal	325 (21.7)	228 (22.3)	57 (19.4)	40 (22.2)	
Post-pubertal	1040 (69.6)	704 (68.9)	205 (69.7)	131 (72.8)	

BMI: body mass index; IQR: interquartile range. Difference of age values between the study groups were compared using the Kruskal–Wallis test. Qualitative variables were represented by frequency and compared using the Pearson’s chi-square test. * Missing data: Socioeconomic status (*n* = 48, 3.2%); Pubertal development (*n* = 5, 0.3%).

**Table 2:** Consumption of food groups according to divergence between measured and perceived weight in grams.

Food or Food Group	Total	Agreed	Underestimated	Overestimated	*p*-Value
Sugar and sweets	243.3 (130.2–436.1)	242 (134.4–426.5)	244.2 (122.6–485.2)	245.7 (132.7–420.5)	0.6203
Sweetened beverages	480.4 (200.0–915.3)	480 (213.3–880)	533.4 (193.3–960.1)	466.7 (160.1–946.9)	0.6436
Processed meat products	11.0 (5.5–33.0)	10.99 (5.5–33)	11 (5.5–33)	11 (5.5–32.9)	0.6126
Fast food	170.4 (80.3–352.7)	165.4 (80.32–339.2)	202.5 (83–419.8)	157 (79.5–323.9)	0.1092
Typical Brazilian dishes	97.3 (49.3–239.8)	96.6 (50–238.3)	112.6 (51.7–246.7)	97.2 (38.3–225.3)	0.2162
Oils	29.3 (11.5–51.1)	29.3 (11.47–50.3)	29.7 (13.9–55)	25.7 (10.7–44.7)	0.0995
Milk and dairy	166.4 (70.9–337.6)	167.6 (73.7–335.9)	175.7 (72.9–386.9)	154.7 (52.7–313.1)	0.2092
Meat	122.7 (64.0–236.7)	117.7 (62.7–225.3)	137.3 (69.3–271.3)	129.3 (62–240)	0.0711
Rice and cereals	460.7 (261.8–730.6)	460.4 (262.2–729.6)	488.8 (282.8–733.8)	439.7 (248.2–753.8)	0.7168
Roots	24.7 (6.8–71.8)	24.7 (6.8–67)	24.7 (3.5–91.2)	27 (3.5–73.3)	0.8940
Beans and legumes	148.8 (78.0–286.0)	154.6 (78–286)	148.8 (56.4–286)	143 (57.8–286)	0.0530
Vegetables	67.3 (23.1–161.2)	66.1 (25.3–164.1)	71.5 (16.1–167.7)	61.5 (22–149.7)	0.5295
Fruits	465.7 (218.3–988.4)	457 (229.5–987.1)	574.2 (205.6–1071)	429.3 (183–848.6)	0.0691
Coffee and tea	106.7 (13.3–293.3)	146.7 (16.7–293.3)	80 (13.3–293.3)	80 (13.3–293.3)	0.4200

Consumption of individual food groups in individuals according to divergence between measured and perceived weight. Data represent median and interquartile range of total consumption of each food group in grams. Distribution of the data in the groups of individuals was compared using the Kruskal–Wallis test.

**Table 3:** Abundance of consumption of each food group in the diet in different dietary patterns.

Food or Food Group	Dietary Patterns				
	Pattern 1	Pattern 2	Pattern 3	Pattern 4	*p*-Value
Sugar and sweets	22.9 ± 18.3	13.6 ± 9.8	18.1 ± 14.1	43.0 ± 39.5	<0.001
Sweetened beverages	49.7 ± 41.2	30.4 ± 24.7	35.7 ± 28.2	137.4 ± 111.9	<0.001
Processed meat products	16.0 ± 21.0	10.5 ± 19.7	12.3 ± 18.1	30.8 ± 32.0	<0.001
Fast food	13.6 ± 13.3	10.2 ± 8.8	11.5 ± 13.4	35.8 ± 35.1	<0.001
Typical Brazilian dishes	24.8 ± 24.6	19.9 ± 10.1	20.5 ± 21.2	63.7 ± 63.1	<0.001
Oils	5.7 ± 5.1	2.2 ± 2.7	4.0 ± 3.3	7.2 ± 8.1	<0.001
Milk and dairy	17.2 ± 17.5	8.4 ± 6.5	15.0 ± 15.0	41.9 ± 40.6	<0.001
Meat	15.7 ± 14.0	9.7 ± 12.9	15.9 ± 20.1	29.8 ± 26.2	<0.001
Rice and cereals	34.1 ± 18.3	14.8 ± 11.2	28.6 ± 16.8	53.2 ± 36.6	<0.001
Roots	11.8 ± 18.3	6.6 ± 7.8	10.3 ± 16.5	27.1 ± 36.9	<0.001
Beans and legumes	82.3 ± 59.1	17.4 ± 15.9	111.6 ± 71.9	104.8 ± 76.3	<0.001
Vegetables	15.2 ± 17.8	8.4 ± 9.3	10.3 ± 10.9	29.0 ± 24.6	<0.001
Fruits	30.5 ± 32.0	17.4 ± 17.7	21.8 ± 18.7	58.5 ± 48.5	<0.001
Coffee and tea	156.2 ± 64.5	10.9 ± 11.2	32.8 ± 31.7	74.2 ± 91.2	<0.001

Data represent mean and standard deviation of values calculated for abundance of consumption of each food or food group (unit = % consumption relative to total diet). The one-way ANOVA test was used to examine the differences in values of abundance of consumption of each indicated food group, relative to total diet, between the dietary patterns identified by the hierarchical cluster analysis presented in Figure 2. The parametric test was used because the distribution of values of abundance of consumption exhibited a Gaussian distribution assessed by the D’Agostino–Pearson test.

## Supplementary Materials

The following are available online at https://www.mdpi.com/2072-6643/12/6/1670/s1. Table S1. Food groups according to similarity in nutritional composition.
