# Supplementary material for: Systems Nutrology of Adolescents with Divergence between Measured and Perceived Weight Uncovers a Distinctive Profile Defined by Inverse Relationships of Food Consumption
Source: Nutrients. 2020 Jun 4;12(6):1670. doi: 10.3390/nu12061670 (PMC7352492; doi:10.3390/nu12061670)
Supplement: Supplementary file 1 [file nutrients-12-01670-s001.pdf]

Table S1. Food groups according to similarity in nutritional composition, consumed by adolescents enrolled in the study

Food or food groups	Food items from the food frequency questionnaire
1. Sugar and sweets	Sugar, chocolate powder, homemade sweets, industrialized sweets, stuffed biscuit, candies, chewing gum, lollipops, chocolate bar, gelatin, ice cream and popsicle (cream and/or chocolate).
2. Sweetened beverages	Normal, diet or light soda, artificial juice, carbonated drinks, artificial refreshment, energy drink and liquid or powdered sweetener.
3. Typical Brazilian dishes	Acarajé and abará[a], vatapá[b], caruru[c], feijoada[d], dobradinha[e], feijão tropeiro[f] and coconut milk.
4. Fast food	Fried potatoes, potato chips, pizza, lasagna, ketchup, ready-made soups, sandwich, industrialized salty snack, instant noodles, ready-to-eat sauce and pizza-ready sauce.
5. Oils	Butter, margarine, vegetable oil, mayonnaise, olive oil, palm oil.
6. Milk and dairy	Whole milk powder or liquid, skimmed milk powder or liquid, fermented milk, yogurt (whole, diet or light), chocolate ready, yellow cheese, white cheese, cream cheese, creamy curd (whole or light).
7. Meat	Bovine (fried or cooked), chicken with or without skin (fried or cooked), cooked or fried fish, seafood, viscera, chicken egg (fried or cooked), dehydrated meat (Jerky beef).
8. Processed meat products	Ham, mortadella, sausage, calabrese.
9. Rice and cereals	Bread (white or whole), rice (white or whole), noodles (white or whole), cassava flour, farinaceous (oats, wheat germ), milk or nest meal, green corn or couscous of corn, popcorn salted), homemade cake, box cake, granola, biscuit (salted or sweet), pasta soup.
10. Roots	Cassava, sweet potato, potato.
11. Beans and legumes	Beans, peanuts, nuts and walnuts.
12. Vegetables	Lettuce, cabbage, cabbage, pumpkin, carrot, tomato, chayote, gherkin, beet, okra, vegetable salad.
13. Fruits	Pineapple, avocado, acerola, silver banana, ground banana, cashew, jackfruit, papaya, mango, apple, watermelon, melon, orange, tangerine, strawberry, fruit juice or fruit pulp, acai-berry
14. Coffee and tea	Coffee and tea

[a] Dishes made of beans and shrimp, deep-fried in palm oil, the only difference is that abará is steamed, while acarajé is fried. [b] Creamy paste prepared with bread, shrimp, coconut milk, finely ground peanuts and palm oil. [c] Made from okra, onion, shrimp, palm oil and toasted nuts (peanut and/or cashew). [d] Stew of beans with beef and pork. [e] Dish made from a cow's flat white stomach lining. [f] Cattleman's Beans.
